# Inhibition of Aflatoxin Production by Paraquat and External Superoxide Dismutase in *Aspergillus flavus*

**DOI:** 10.3390/toxins11020107

**Published:** 2019-02-12

**Authors:** Tomohiro Furukawa, Shohei Sakuda

**Affiliations:** Department of Biosciences, Teikyo University, 1-1 Toyosatodai, Utsunomiya 320-8551, Japan; furukawa@nasu.bio.teikyo-u.ac.jp

**Keywords:** aflatoxin production, *Aspergillus flavus*, superoxide, paraquat, superoxide dismutase

## Abstract

Aflatoxin contamination of crops is a worldwide problem, and elucidation of the regulatory mechanism of aflatoxin production, for example relative to the oxidative–antioxidative system, is needed. Studies have shown that oxidative stress induced by reactive oxygen species promotes aflatoxin production. However, superoxide has been suggested to have the opposite effect. Here, we investigated the effects of the superoxide generator, paraquat, and externally added superoxide dismutase (SOD) on aflatoxin production in *Aspergillus flavus*. Paraquat with an IC_50_ value of 54.9 µM inhibited aflatoxin production without affecting fungal growth. It increased cytosolic and mitochondrial superoxide levels and downregulated the transcription of aflatoxin biosynthetic cluster genes, including *aflR*, a key regulatory protein. The addition of bovine Cu/ZnSOD to the culture medium suppressed the paraquat-induced increase in superoxide levels, but it did not fully restore paraquat-inhibited aflatoxin production because bovine Cu/ZnSOD with an IC_50_ value of 17.9 µg/mL itself inhibited aflatoxin production. Externally added bovine Cu/ZnSOD increased the SOD activity in fungal cell extracts and upregulated the transcription of genes encoding Cu/ZnSOD and alcohol dehydrogenase. These results suggest that intracellular accumulation of superoxide impairs aflatoxin production by downregulating *aflR* expression, and that externally added Cu/ZnSOD also suppresses aflatoxin production by a mechanism other than canonical superoxide elimination activity.

## 1. Introduction

Aflatoxins are potent carcinogenic toxins produced mainly by *Aspergillus flavus* and *Aspergillus parasiticus*, which infect agricultural crops, including corn and peanut. Aflatoxins accumulated in crops cause mycotoxicosis in humans and domestic animals that ingest them [1,2]. Crops contaminated with aflatoxins are discarded or reduced in value, resulting in significant economic losses [3]. Many challenges are involved in the control of aflatoxin contamination; the molecular mechanism to regulate aflatoxin production level must be elucidated to optimize or develop effective preventive methods [4,5,6].

The enzymatic genes responsible for aflatoxin biosynthesis are located in a cluster in the genomes of *A. flavus* and *A. parasiticus*. Aflatoxins are biosynthesized from 10 acetic acid units via at least 18 reaction steps [7]. The transcription of genes encoding aflatoxin biosynthetic enzymes is positively regulated by the transcription factor AflR, whose gene is in the same cluster [8]. Environmental factors such as light and pH, trophic factors such as carbon and nitrogen sources, and several transcription factors recognizing these cues have been found to affect aflatoxin production, but the regulatory mechanisms of these factors leading to aflatoxin production have not been clarified in detail [9,10,11,12].

As externally added hydrogen peroxide has been found to promote aflatoxin production in *A. parasiticus* and *A. flavus*, the relationship between aflatoxin production and oxidative stress caused by reactive oxygen species (ROS) has received wide attention [13,14,15]. ROS comprise a series of molecular species with high chemical reactivity generated from oxygen. As ROS react with macromolecules such as DNA, protein, and lipids, impairing their function, cells are equipped with antioxidant systems that protect biomolecules from ROS [16]. When the balance between antioxidant systems and ROS generation is disrupted, oxidative stress occurs. In fungal cells, superoxide, a byproduct of the mitochondrial electron transport chain, is the main source of intracellular ROS [17]. The superoxide generated is decomposed into hydrogen peroxide and oxygen by superoxide dismutase (SOD), and hydrogen peroxide is decomposed into water by antioxidant enzymes, including catalase, glutathione peroxidase, and peroxiredoxin. Free Fe^2+^ may react with hydrogen peroxide and produce hydroxyl radical, which is highly toxic due to its high reactivity [18].

Although excessive ROS are harmful, ROS adjusted to an appropriate level function as signaling molecules in cell proliferation and differentiation [19]. In *A. parasiticus* aflatoxigenic strain NRRL2999, oxygen consumption increased in the logarithmic growth phase, and the enzymatic activities of SOD and glutathione peroxidase increased synchronously with aflatoxin production [20]. However, these phenomena of oxygen consumption and antioxidant enzyme activities observed in the aflatoxigenic strain were not observed in the nontoxigenic SRRC255 strain, suggesting that elevated ROS levels due to an increase in oxygen uptake are correlated with aflatoxin production and the expression of antioxidant enzymes. Hydrogen peroxide increased aflatoxin production in *A. flavus* NRRL3357 in a concentration-dependent manner [14]. Antioxidants and thiol redox state modulators reduced aflatoxin production in the *A. flavus* 70S(pSL82) strain [21]. These observations suggest that a decrease in the ROS level causes a decrease in aflatoxin production. On the other hand, Zaccaria et al. [22] indicated that menadione, a superoxide generator, suppressed aflatoxin production in *A. flavus* NRRL3357, accompanied by a decrease in SOD activity. The regulation of mycotoxin production by superoxide was also observed in *Fusarium graminearum*, which accumulates trichothecenes in infected grains. The superoxide generator paraquat reduced trichothecene production in several strains [23,24]. Furthermore, in SOD gene deletion mutants of *F. graminearum*, the accumulation of intracellular superoxide and reduction of trichothecene production were observed [25].

As ROS differ in terms of generation and elimination characteristics in fungal cells, as well as chemical reactivity, the effects of individual ROS on aflatoxin production must be investigated in detail to understand the regulatory mechanism of aflatoxin production by ROS. In this study, we focused on superoxide and evaluated the effects of paraquat and external SOD on aflatoxin production in *A. flavus*. We obtained paradoxical results; both paraquat and SOD suppressed aflatoxin production. In this paper, we describe the effects of superoxide generated from paraquat on mitochondrial function, reducing *aflR* expression, and the apparent partial internalization of external SOD into cells to suppress aflatoxin production, possibly by a function other than superoxide dismutation activity.

## 2. Results

### 2.1. Effect of Paraquat on Aflatoxin Production

**Figure 1:**
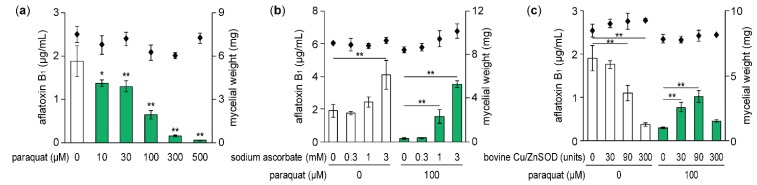
Effects of paraquat, sodium ascorbate, and Cu/Zn superoxide dismutase (Cu/ZnSOD) on aflatoxin B_1_ production and fungal growth of *A. flavus*. (**a**–**c**) The amount of aflatoxin B_1_ (white bars, without paraquat; green bars, with paraquat) and mycelial dry weight (squares) were analyzed. Data are presented as means and standard deviations from three biological replicates. Asterisks indicate significant differences (* *P* < 0.05, ** *P* < 0.01 vs. control group, Dunnett test).

When *A. flavus* IFM 47798 was incubated for 48 h at 28 °C in potato dextrose broth (PDB) liquid medium, about 1–2 ppm aflatoxin B_1_ was detected in the culture broth. The amount of aflatoxin B_1_ produced by the strain decreased in a concentration-dependent manner by addition of paraquat with the IC_50_ value of 54.9 μM (Figure 1a). As the fungal mycelial dry weight was not changed significantly by 500 μM paraquat, the inhibitory activity of this superoxide generator was specific to aflatoxin B_1_ production. The inhibition of aflatoxin B_1_ production by paraquat was thought to be due to the generation of intracellular superoxide. Therefore, we examined whether the effect of paraquat was affected by sodium ascorbate, a general antioxidant. Aflatoxin B_1_ production suppressed by 100 μM paraquat was significantly restored by co-addition of >1 mM sodium ascorbate (Figure 1b). Furthermore, the addition of 3 mM sodium ascorbate without paraquat significantly promoted aflatoxin B_1_ production.

### 2.2. Effect of External SOD on Aflatoxin Production

Next, we examined whether externally added SOD could affect the inhibition of aflatoxin production by paraquat. *A. flavus* was cultured with bovine Cu/ZnSOD (30, 90, and 300 units/2 mL culture) and/or paraquat, and the amount of aflatoxin B_1_ produced was measured (Figure 1c). In cultures with 100 μM paraquat, aflatoxin B_1_ production was restored to some extent by 30 and 90 units of Cu/ZnSOD compared with no Cu/ZnSOD, but the small amount of aflatoxin production caused by paraquat was not changed by 300 units of Cu/ZnSOD. On the other hand, in cultures without paraquat, the amount of aflatoxin B_1_ was decreased in a concentration-dependent manner by Cu/ZnSOD with an IC_50_ value of 107.3 units, corresponding to 17.9 μg protein/mL. These results suggest that externally added Cu/ZnSOD could decrease the amount of intracellular superoxide generated by paraquat, leading to the partial recovery of aflatoxin B_1_ production. However, 300 units of Cu/ZnSOD could not suppress the effect of paraquat because its inhibitory activity on aflatoxin production was sufficiently strong to reduce the amount of aflatoxin to the level observed in the culture with 100 μM paraquat alone.

### 2.3. Effects of Paraquat and External SOD on mRNA Levels of Genes Responsible for Aflatoxin Biosynthesis

**Figure 2:**
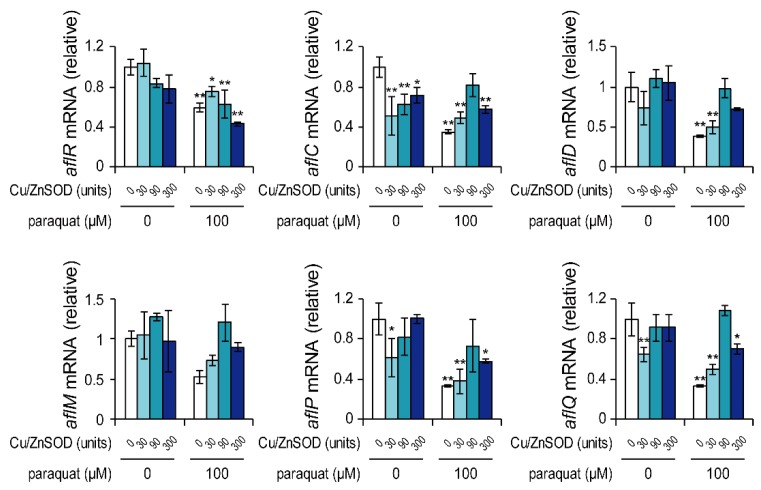
Effects of paraquat and Cu/ZnSOD on the mRNA levels of aflatoxin cluster genes. Transcription of each gene was analyzed by real-time quantitative PCR. Each mRNA level was normalized to the amount of *β-tubulin* mRNA in each sample. Data are presented as means and standard deviations from three biological replicates. Asterisks indicate significant differences (* *P* < 0.05, ** *P* < 0.01 vs. control group, Dunnett test).

*A. flavus* was cultured for 48 h with paraquat and/or Cu/ZnSOD, and mRNA levels of genes in the aflatoxin biosynthetic gene cluster were examined by real-time PCR (Figure 2). In the culture with 100 μM paraquat, the mRNA levels of *aflR* and four genes encoding biosynthetic enzymes (AflC, AflD, AflP, and AflQ) were significantly decreased compared with the control, suggesting that the inhibition of aflatoxin B_1_ production by paraquat was due to suppressed transcription of the aflatoxin cluster genes. The co-addition of Cu/ZnSOD to the culture with 100 μM paraquat recovered the mRNA levels of these genes to some extent, but these levels remained lower than those in the control group. In general, addition of Cu/ZnSOD alone did not affect the mRNA levels of these genes, with the exception of *aflC*. These results suggest that Cu/ZnSOD inhibited aflatoxin B_1_ production without significantly affecting the transcription of most aflatoxin biosynthetic cluster genes.

### 2.4. Effects of External SOD on mRNA Levels of Genes Encoding SOD and Acetyl-CoA Metabolic Enzymes

**Figure 3:**
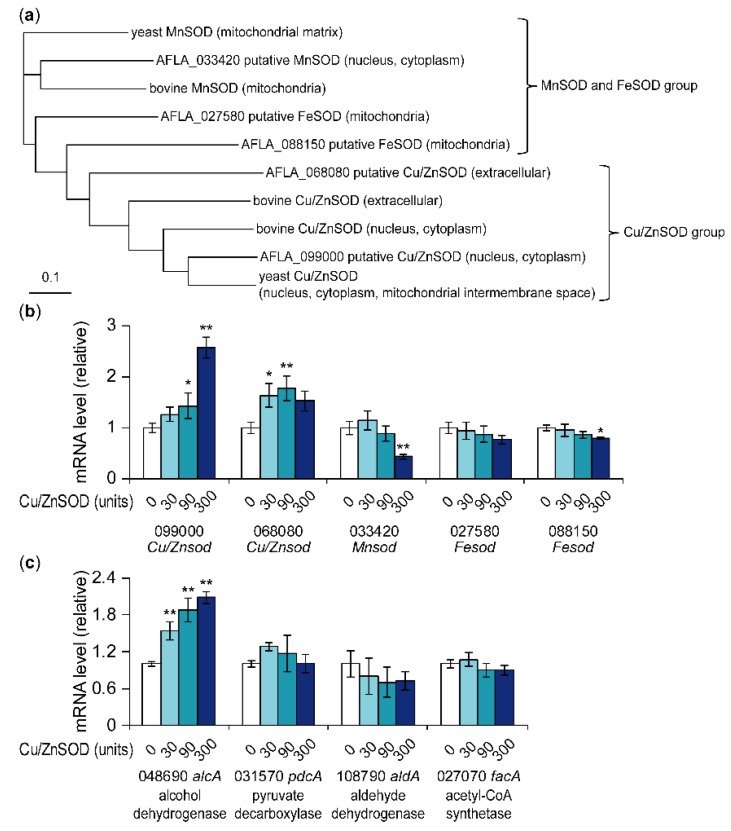
Phylogenetic analysis of *A. flavus* putative SOD genes and effects of Cu/ZnSOD on the mRNA levels of these genes. (**a**) Amino acid sequences of five *A. flavus* putative SODs, two yeast SODs, and three bovine SODs were aligned using the clustal omega algorithm (provided on the website of European Bioinformatics Institute [30]). The phylogenetic tree was constructed using the neighbor-joining method. The localization of *A. flavus* SODs was predicted by the TargetP 1.1 server [27]. The localization of yeast and bovine SODs was predicted following the description on the UniProt website [31]. (**b**,**c**) Transcription of each gene was analyzed by real-time quantitative PCR. The amount of each mRNA was normalized to the amount of *β-tubulin* mRNA in each sample. Data are presented as means and standard deviations from three biological replicates. Asterisks indicate significant differences (* *P* < 0.05, ** *P* < 0.01 vs. control group, Dunnett test).

In the genome of *A. flavus* NRRL3357, five genes were annotated as SOD genes. From the multiple alignment of amino acid sequences of the five genes (AFLA_099000, AFLA_068080, AFLA_033420, AFLA_027580, and AFLA_088150), two yeast SODs (yeast MnSOD and yeast Cu/ZnSOD [26]), and three bovine SODs (one MnSOD and two Cu/ZnSODs), a phylogenetic tree was created (Figure 3a). Cellular localization of the five *A. flavus* SODs was predicted using the TargetP 1.1 server (Figure 3a) [27]. AFLA_099000 was closest to yeast Cu/ZnSOD and was predicted to be localized to the cytoplasm or nucleus. AFLA_068080 was predicted to be extracellular Cu/ZnSOD. AFLA_033420 was closest to yeast mitochondrial MnSOD and was predicted to be localized to the cytoplasm or nucleus. AFLA_027580 and AFLA_088150 were predicted to be localized to the mitochondria and annotated as FeSOD. As Fe-type SODs can utilize Fe and Mn as metal cofactors [28], which metal is utilized by these SODs is unclear. Real-time PCR analysis showed that the addition of Cu/ZnSOD significantly increased the mRNA levels of AFLA_099000 putative Cu/ZnSOD and AFLA_068080 putative Cu/ZnSOD (Figure 3b). Conversely, the addition of 300 units Cu/ZnSOD significantly decreased the mRNA levels of AFLA_033420 putative MnSOD and AFLA_088150 putative FeSOD. These results suggest that the external addition of Cu/ZnSOD affected the intranuclear transcriptional regulation of the fungal intrinsic SODs.

In the early stage of aflatoxin biosynthesis in peroxisomes, the biosynthetic precursor acetyl-CoA could be supplied from β-oxidation in peroxisomes and mitochondria and/or acetate generated from acetaldehyde [29]. Therefore, acetaldehyde may be a key metabolite for aflatoxin production. Acetaldehyde, which is produced from pyruvate by pyruvate decarboxylase, is converted to ethanol by alcohol dehydrogenase or to acetate by aldehyde dehydrogenase. Acetate is further converted to acetyl-CoA by acetyl-CoA synthetase. Real-time PCR conducted to analyze the effect of Cu/ZnSOD on the mRNA levels of four genes encoding pyruvate decarboxylase, alcohol dehydrogenase, aldehyde dehydrogenase, and acetyl-CoA synthetase indicated that Cu/ZnSOD significantly increased the mRNA level of AFLA_048690 putative alcohol dehydrogenase in a concentration-dependent manner (Figure 3c). This result suggests that Cu/ZnSOD increased acetaldehyde-derived ethanol, which in turn decreased acetyl-CoA, leading to the repression of aflatoxin production.

### 2.5. SOD Activities in Fungal Cells

**Figure 4:**
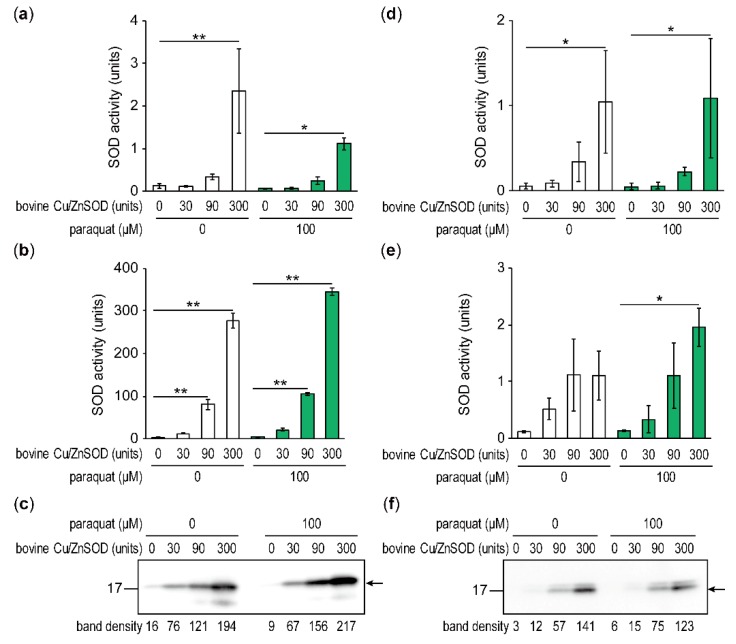
Determination of SOD activity and SOD protein abundance. (**a**–**c**) *A. flavus* was cultured for 48 h. SOD activity of fungal cell extracts (**a**) and culture supernatants (**b**) was determined, and fungal cell extracts were subjected to western blotting using anti-human SOD1 antibody (**c**). The density of the bands indicated by the arrow was quantified using Image J. (**d**–**f**) After *A. flavus* was cultured for 24 h, mycelia were collected, washed with distilled water, transferred to fresh medium, and incubated for another 24 h. Then SOD activity in fungal cell extracts (**d**) and culture supernatant (**e**) was determined. Fungal cell extracts were subjected to western blotting using anti-human SOD1 antibody, and the density of the bands indicated by the arrow was quantified (**f**). Data are presented as means and standard deviations from three biological replicates. Asterisks indicate significant differences (* *P* < 0.05, ** *P* < 0.01 vs. control group, Dunnett test).

To investigate whether externally added bovine Cu/ZnSOD altered intracellular SOD activities, SOD activities in the cell extracts and culture supernatant were measured. Regardless of the presence or absence of paraquat, SOD activity was significantly greater in fungal cell extracts cultured with 300 units of Cu/ZnSOD for 48 h than in cultures without Cu/ZnSOD (Figure 4a). About 0.5–1% of the SOD activity at the beginning of cultivation was detected in the fungal cell extracts; most SOD activity remained in the culture supernatant (Figure 4b), indicating that bovine Cu/ZnSOD added to the culture remained undegraded. Next, the protein abundance of SOD was examined by western blotting of the cell extracts using an anti-human SOD1 antibody, which has cross-reactivity with bovine nucleic and cytoplasmic 15.7-kDa Cu/ZnSOD. Western blotting revealed bands around 17 kDa, even without the addition of bovine Cu/ZnSOD, suggesting that 16-kDa AFLA_099000 putative Cu/ZnSOD was also detected by anti-human SOD1 antibody (Figure 4c). The density of the bands detected depended on the Cu/ZnSOD activity depicted in Figure 4a.

To show that SOD activity was maintained in the mycelia, mycelia of *A. flavus* cultured for 24 h were collected, washed with water three times, and transferred to fresh medium. After cultivation in the fresh medium for another 24 h, SOD activities in the mycelia and supernatant were measured. With and without paraquat, about 1–2 units of SOD activity were observed in each cell extract and the supernatant cultured with 300 units of Cu/ZnSOD before washing of the mycelia (Figure 4d–f), suggesting that elevated SOD activity in fungal cells were maintained after 24 h cultivation.

### 2.6. Effects of Paraquat and External SOD on Mitochondrial and Cytosolic Superoxide Levels

**Figure 5:**
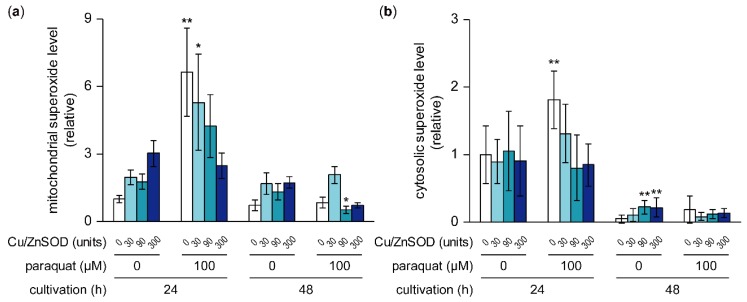
Effects of paraquat and Cu/ZnSOD on mitochondrial and cytosolic superoxide levels. Time courses of mitochondrial (**a**) and cytosolic (**b**) superoxide levels in *A. flavus* were calculated by analyzing mitoSOX and dihydroethidium fluorescence, respectively, on microscopic images. Data are presented as means and standard deviations from eight or more microscopic images. Asterisks indicate significant differences (* *P* < 0.05, ** *P* < 0.01 vs. control group, Dunnett test).

To estimate how the intracellular superoxide level was affected by the addition of paraquat and/or Cu/ZnSOD, cellular superoxide was detected using the superoxide-specific fluorescent indicators mitoSOX and dihydroethidium (DHE), which are localized to the mitochondria and cytoplasm, respectively (Figure 5a,b and Supplementary Figure S1). In cultures treated with 100 μM paraquat alone, superoxide levels in the mitochondria and cytoplasm were significantly higher than in the control after 24 h cultivation. These paraquat-induced high superoxide levels were suppressed by the addition of Cu/ZnSOD in a concentration-dependent manner. In contrast, in cultures without paraquat, neither the mitochondrial nor the cytoplasmic superoxide level was changed significantly by the addition of Cu/ZnSOD at 24 h cultivation. These results suggest that the external addition of Cu/ZnSOD led to the decomposition of superoxide, the level of which had been increased by paraquat, whereas it did not affect the superoxide level during normal fungal growth.

## 3. Discussion

In some aflatoxigenic strains, aflatoxin production has been reported to be influenced by ROS; hydrogen peroxide promotes aflatoxin production, and ROS regulators such as antioxidants and intracellular ROS generator suppress it [12,13,14,15,20,21]. However, the manner in which individual ROS intrinsically regulate aflatoxin production needs to be clarified. In this study, we confirmed that paraquat increased superoxide levels in the mitochondria and cytoplasm, and inhibited aflatoxin B_1_ production by suppressing the expression of aflatoxin biosynthetic cluster genes, including *aflR*. In contrast to previous reports on the pSL82 strain, we found that ascorbate promoted aflatoxin B_1_ production during the experiment with paraquat [21]. When bovine Cu/ZnSOD was used for the dismutation of paraquat-induced superoxide, we observed that Cu/ZnSOD suppressed aflatoxin B_1_ production in a concentration-dependent manner.

The addition of bovine Cu/ZnSOD not only increased SOD activity in fungal cell extracts, but also increased the mRNA level of AFLA_099000 putative Cu/ZnSOD. Therefore, it was difficult to determine whether bovine Cu/ZnSOD was internalized to increase SOD activity in the fungal cell extracts. Peñalva [32] reported that fluorescent dye FM4-64 was internalized into the cells of *Aspergillus nidulans* in energy-, temperature-, and F-actin–dependent manners. The fluorescence of FM4-64 was detected, in order, in cortical organelles (e.g., actin-patch), hollow structures with diameters of 0.7 µm representing mature endosomes, and 2–3-µm-diameter vacuoles, leading the author to conclude that FM4-64 was internalized by endocytosis. Higuchi et al. [33] found that plasma membrane protein AoUapC-EGFP, the fusion protein of a putative uric acid–xanthine permease with enhanced green fluorescent protein, was internalized into the cells of *Aspergillus oryzae* upon the addition of ammonium. As the internalization was temperature and F-actin dependent, the authors concluded that this membrane protein was internalized by endocytosis. Based on these reports, bovine Cu/ZnSOD could be internalized by endocytosis with other extracellular nutrients.

Increase in mitochondrial superoxide is thought to be a major cause of inhibition of aflatoxin B_1_ production by paraquat. Paraquat generates superoxide by oxidation of the paraquat radical, which is generated via one-electron reduction by respiratory chain or other dehydrogenases in the mitochondria [34]. Generated superoxide attacks mitochondrial (4Fe-4S) cluster enzymes, including aconitase, and releases Fe^2+^ from the cluster to inactivate the enzyme [35,36]. This Fe^2+^ release leads to the generation of hydroxyl radical, a strong oxidizing agent of macromolecules [18]. Therefore, paraquat may cause mitochondrial dysfunction. As mitochondrial respiratory inhibitors inhibit aflatoxin production [37], paraquat probably inhibits aflatoxin production through mitochondrial dysfunction. The relationship between mitochondrial function and the transcriptional regulation of aflatoxin biosynthetic cluster genes will be subject to further investigation.

Fluorescence observation indicated that paraquat increased mitochondrial and cytosolic superoxide, possibly through the flow of excess superoxide from the mitochondria into the cytosol through membrane channels, such as the voltage-dependent anion channel (VDAC) [17]. The addition of bovine Cu/ZnSOD suppressed the paraquat-induced superoxide elevation in the mitochondria and cytosol, probably by increasing SOD activity.

Inhibition of aflatoxin B_1_ production by bovine Cu/ZnSOD may occur due to the function of Cu/ZnSOD as a transcription factor affecting expression of oxidative response genes. As the addition of bovine Cu/ZnSOD without paraquat did not significantly change the mitochondrial or cytoplasmic superoxide level, the inhibition of aflatoxin production by Cu/ZnSOD may not be correlated with SOD’s canonical superoxide scavenging function. Tsang et al. [38] showed that SOD1 in human and yeast cells is phosphorylated by the Mec1/ATM kinase cascade in response to intracellular hydrogen peroxide, and that the phosphorylated SOD1 is translocated into the nucleus, where it binds to the promoters of oxidative stress–responsive genes and promotes their transcription. Yeast *por-1* encodes VDAC1, and growth of its disruptant is restricted under non-fermentable carbon source. Magrì et al. [39] found that overexpression of human SOD1 restored the growth of the disruptant in the presence of glycerol as a single carbon source. They concluded that human SOD1 entered the nucleus and increased transcription of the *por-2* gene, which encodes the mitochondrial outer membrane β-barrel VDAC2, resulting in the partial recovery of mitochondrial outer membrane function. If bovine Cu/ZnSOD is internalized, it may be transferred to the nucleus, where it would control the transcription of oxidative stress–responsive genes, including AFLA_099000 encoding cytosolic Cu/ZnSOD. If bovine Cu/ZnSOD is not internalized, the way in which it increases the mRNA levels of AFLA_099000 in a dose-dependent manner is not clear, but the SOD increase caused by bovine Cu/ZnSOD might affect the transcription of some genes.

The addition of bovine Cu/ZnSOD significantly increased transcription of the alcohol dehydrogenase gene. This increased alcohol dehydrogenase activity might promote the conversion of acetaldehyde to ethanol, resulting in decreased acetyl-CoA and aflatoxin B_1_ production. Work investigating whether bovine Cu/ZnSOD enters the nucleus and binds to the promoter regions of some *A. flavus* genes is now in progress. 

## 4. Conclusions

This study clarified that paraquat induces intracellular accumulation of superoxide, affects *aflR* expression, and reduces aflatoxin production. Externally added bovine Cu/ZnSOD suppressed the paraquat-induced increase in superoxide levels and partially restored aflatoxin production. However, bovine Cu/ZnSOD itself can inhibit aflatoxin production by a mechanism other than its superoxide elimination activity.

## 5. Materials and Methods

### 5.1. Chemicals

Aflatoxin B_1_ standard, paraquat, sodium ascorbate, and lyophilized powder of Cu/ZnSOD from bovine erythrocytes were purchased from Sigma-Aldrich (#S7571; St Louis, MO, USA). MitoSOX and DHE were purchased from Thermo Fisher Scientific (Waltham, MA, USA). Paraquat and sodium ascorbate were dissolved in water to be 100 mM and 3 M, respectively. Bovine Cu/ZnSOD was dissolved in phosphate buffered saline (pH7.4) to be 10 U/μL. According to Sigma-Aldrich, one unit will inhibit reduction of cytochrome c by 50% in a coupled system with xanthine oxidase at pH 7.8 at 25 °C in a 3.0 mL reaction volume. Enzyme concentration is about 3000 units/mg protein.

### 5.2. Strain and Culture Conditions

*Aspergillus flavus* IFM 47798 strain (obtained from Agricultural research service, USDA, USA), which mainly produces aflatoxin B_1_, was used throughout this study. A glycerol solution suspending spores collected from a week-old culture plate was used as an inoculum. The spore suspension was inoculated into PDB (BD, Sparks, MD, USA) liquid medium in a 12-well microplate (2 mL/well) at 10^5^ spores/well, and the microplate was placed at 28 °C in the dark for 48 h. When adding paraquat or sodium ascorbate, 2 μL of diluted solution was added in 2 mL culture. When adding bovine Cu/ZnSOD, 30 μL of diluted solution was added. When replacing the culture medium at 24 h of cultivation, the culture broth of each well was centrifuged to obtain mycelia. After mycelia were washed three times with distilled water, mycelia were transferred into 12-well microplate in which each well was filled with 2 mL of fresh PDB liquid medium. Then the plate was placed at 28 °C for another 24 h.

### 5.3. Analysis of Aflatoxin B_1_ Production and Mycelial Weight

After incubation, the culture broth of each well was centrifuged to obtain culture supernatant and mycelia. The 500 μL of supernatant was extracted with 500 μL of chloroform and the chloroform solution was evaporated in the air. The remaining residue was dissolved in 100 μL of 90% aqueous acetonitrile and subjected to reverse-phase HPLC analysis according to the method previously reported [37]. The mycelia were washed with distilled water and lyophilized. The dried mycelia were weighed. 

### 5.4. Determination of SOD Activity

Lyophilized mycelia were ground under liquid nitrogen with mortar and pestle and homogenate was suspended in 200 μL of assay buffer (150 mM sucrose, 20 mM Tris-HCl (pH 8.0), 1 mM EDTA, 0.1% nonidet P-40). The suspension was centrifuged and supernatant was diluted to 1/25 in PBS and its SOD activity was determined using SOD assay kit-WST (Dojindo, Kumamoto, Japan) according to the manufacturer’s instructions. For the culture supernatants, SOD activity was similarly determined using 20 μL of culture supernatants.

### 5.5. Western Blotting

Ten microliters of supernatants of mycelial homogenate suspension described above was subjected to SDS-PAGE and the separated proteins on the gel were transferred to polyvinylidene difluoride (PVDF) membrane. The membrane was immuno-blotted using anti-SOD1 antibody (SPC-115C; StressMarq Biosciences, British Columbia, Canada) followed by goat anti-rabbit IgG (H+L) poly-horseradish peroxidase antibody (32260; Thermo Fisher Scientific). The membrane was developed by ECL prime western blotting detection reagent (GE healthcare, Buckinghamshire, UK) and detected with ChemiDoc XRS+ system (Bio-Rad, Hercules, CA, USA). The density of the bands was relatively quantified using Image J software (US National Institutes of Health, Bethesda, MD, USA).

### 5.6. RT-qPCR Analysis

Lyophilized mycelia were ground as described above. Total RNA was extracted by TRIzol reagent (Thermo Fisher Scientific) and purified using PureLink RNA Mini Kit (Thermo Fisher Scientific). Complementary DNA was synthesized with ReverTra Ace qPCR Master Mix (Toyobo, Osaka, Japan). RT-qPCR was carried out using FastStart Universal SYBR Green Master (Rox) (Roche, Basel, Switzerland) in a final volume of 25 μL for each reaction and ABI PRISM 7300 thermal cycler (Thermo Fisher Scientific). The amount of each mRNA was normalized to the amount of *β-tubulin* (NCBI gene symbol: AFLA_068620) mRNA in each sample. PCR primers used were listed in Supplementary Table S1.

### 5.7. Determination of Superoxide Level

Mitochondrial and cytosolic superoxide levels were quantified in the same manner as previously reported method [25] with some modifications. *A. flavus* was cultured for 24 or 48 hand mycelia were harvested by filtration, washed with distilled water, and incubated with 5 μM mitoSOX or 30 μM DHE for the detection of superoxide in mitochondria and cytosol, respectively. Then the mycelia were incubated with 3 μM Calcofluor White M2R (Sigma-Aldrich) and applied to microscopic slides. A BX53 fluorescence microscope equipped with a DP70 camera (Olympus, Tokyo, Japan) was used to capture fluorescent images. Superoxide level in a region of interest was estimated as follows: Using Image J software, the blue component of each fluorescent image of Calcofluor White M2R was binarized and the dimensions of the binarized area were regarded as mycelial mass in the image. Similarly, the red component of each fluorescent image of mitoSOX and DHE was first subjected to background subtraction and then binarized at the threshold set at “20”. The dimensions of the binarized area were regarded as superoxide amount in the image. The relative superoxide level was calculated with the equation: superoxide level in the image = superoxide amount/mycelial mass × 100. Supplementary Figure S2 shows schematic representation of quantification of superoxide in a region of interest.

### 5.8. Statistical Analysis

Data are presented with the mean ± standard deviation (SD). Differences between groups were analyzed by one-way ANOVA followed by the Dunnett test. Values of *P* < 0.05 were considered to be significant.

## Supplementary Materials

The following are available online at http://www.mdpi.com/2072-6651/11/2/107/s1, Figure S1: Representative fluorescence microscopic images of fungi double-stained by Calcofluor White M2R and superoxide indicator. Figure S2: Schematic representation of quantification of superoxide in a region of interest. Table S1: Primers used in the real-time PCR analysis.
