# Supplementary material for: Inhibition of Aflatoxin Production by Paraquat and External Superoxide Dismutase in Aspergillus flavus
Source: Toxins (Basel). 2019 Feb 12;11(2):107. doi: 10.3390/toxins11020107 (PMC6409742; doi:10.3390/toxins11020107)
Supplement: Supplementary file 1 [file toxins-11-00107-s001.zip › toxins-436906 SPM final.pptx]

## Slide 1
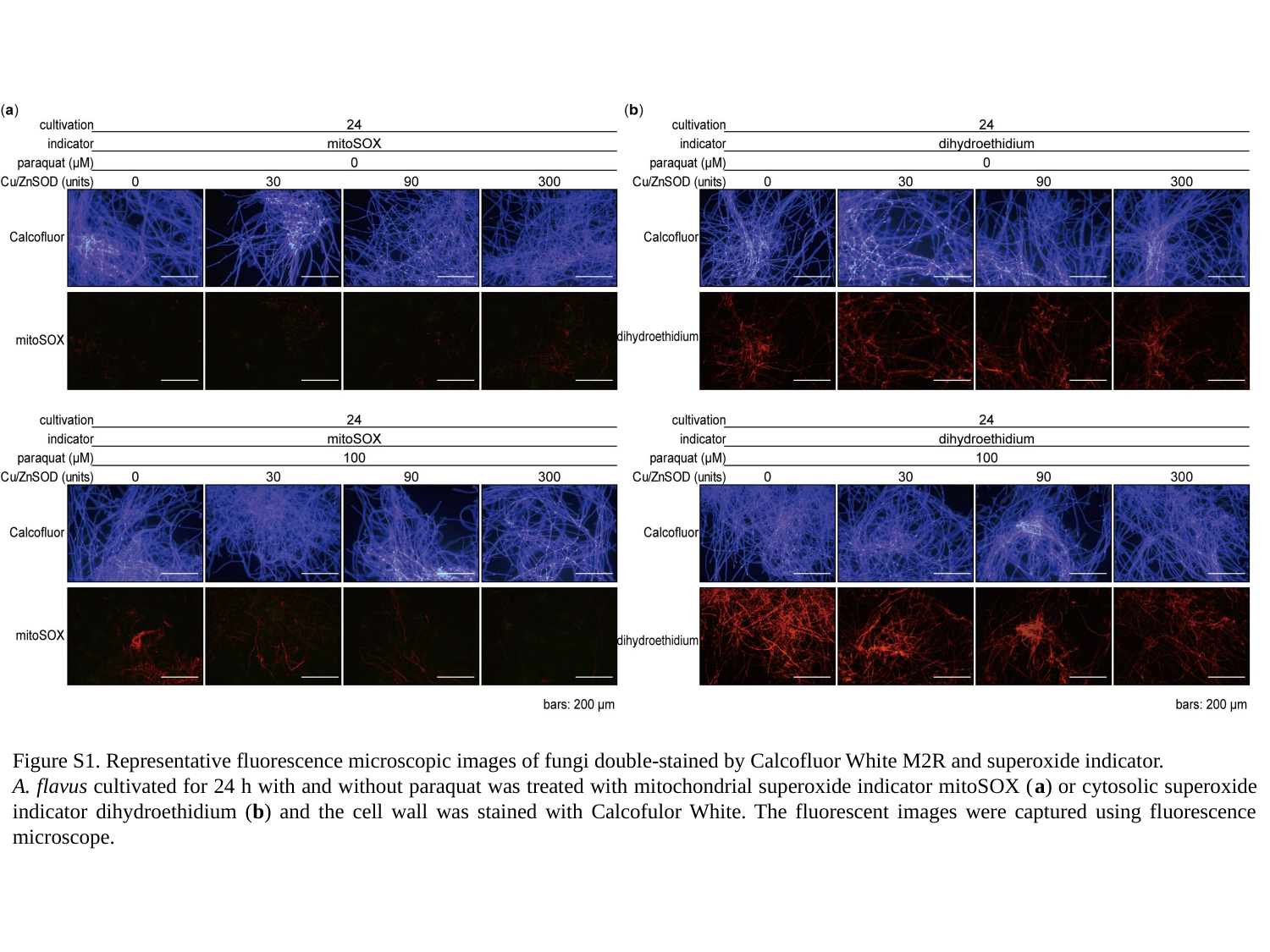

Figure S1. Representative fluorescence microscopic images of fungi double-stained by Calcofluor White M2R and superoxide indicator.
A. flavus cultivated for 24 h with and without paraquat was treated with mitochondrial superoxide indicator mitoSOX (a) or cytosolic superoxide indicator dihydroethidium (b) and the cell wall was stained with Calcofulor White. The fluorescent images were captured using fluorescence microscope.

## Slide 2
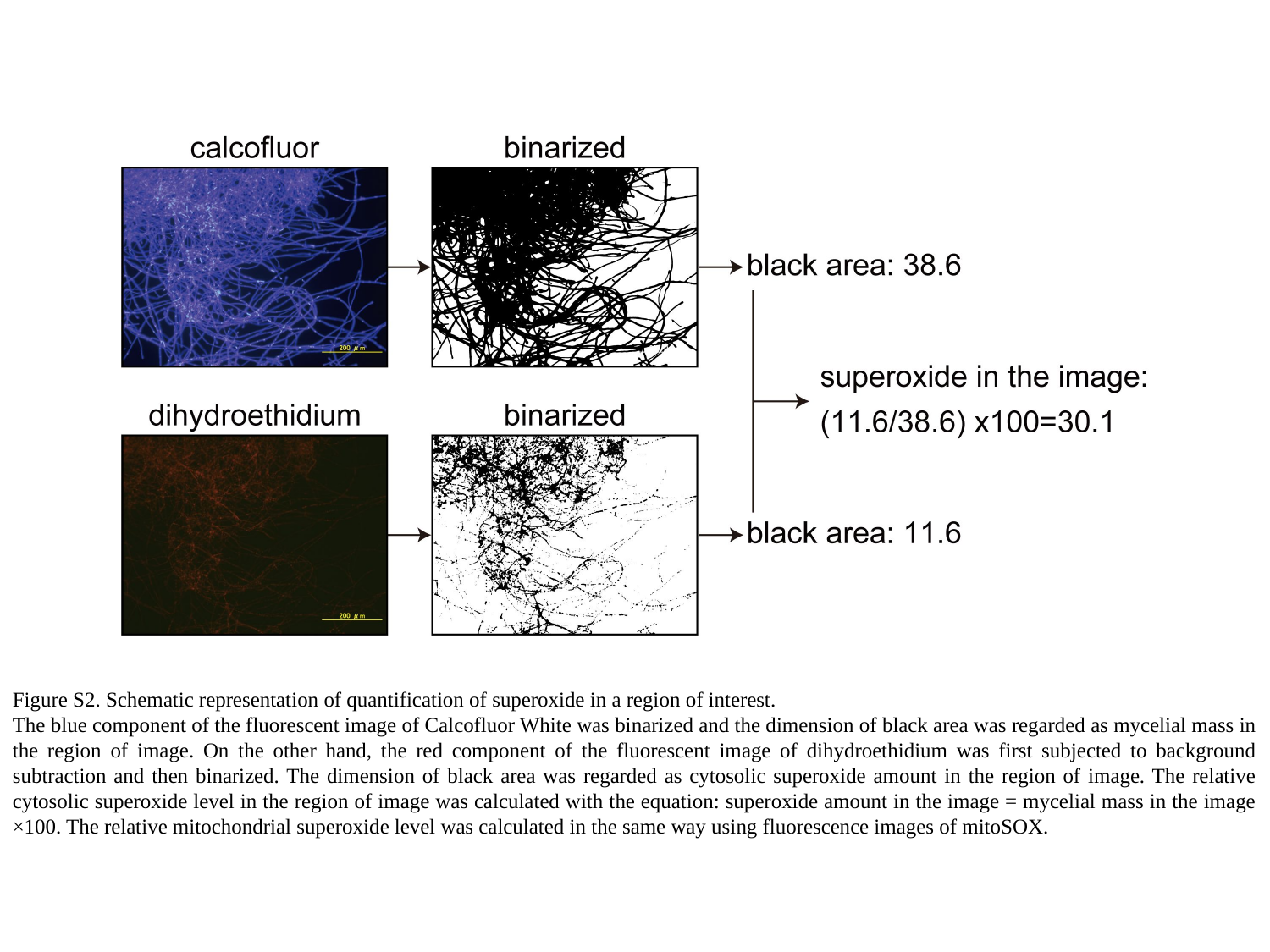

Figure S2. Schematic representation of quantification of superoxide in a region of interest.
The blue component of the fluorescent image of Calcofluor White was binarized and the dimension of black area was regarded as mycelial mass in the region of image. On the other hand, the red component of the fluorescent image of dihydroethidium was first subjected to background subtraction and then binarized. The dimension of black area was regarded as cytosolic superoxide amount in the region of image. The relative cytosolic superoxide level in the region of image was calculated with the equation: superoxide amount in the image = mycelial mass in the image ×100. The relative mitochondrial superoxide level was calculated in the same way using fluorescence images of mitoSOX.

## Slide 3
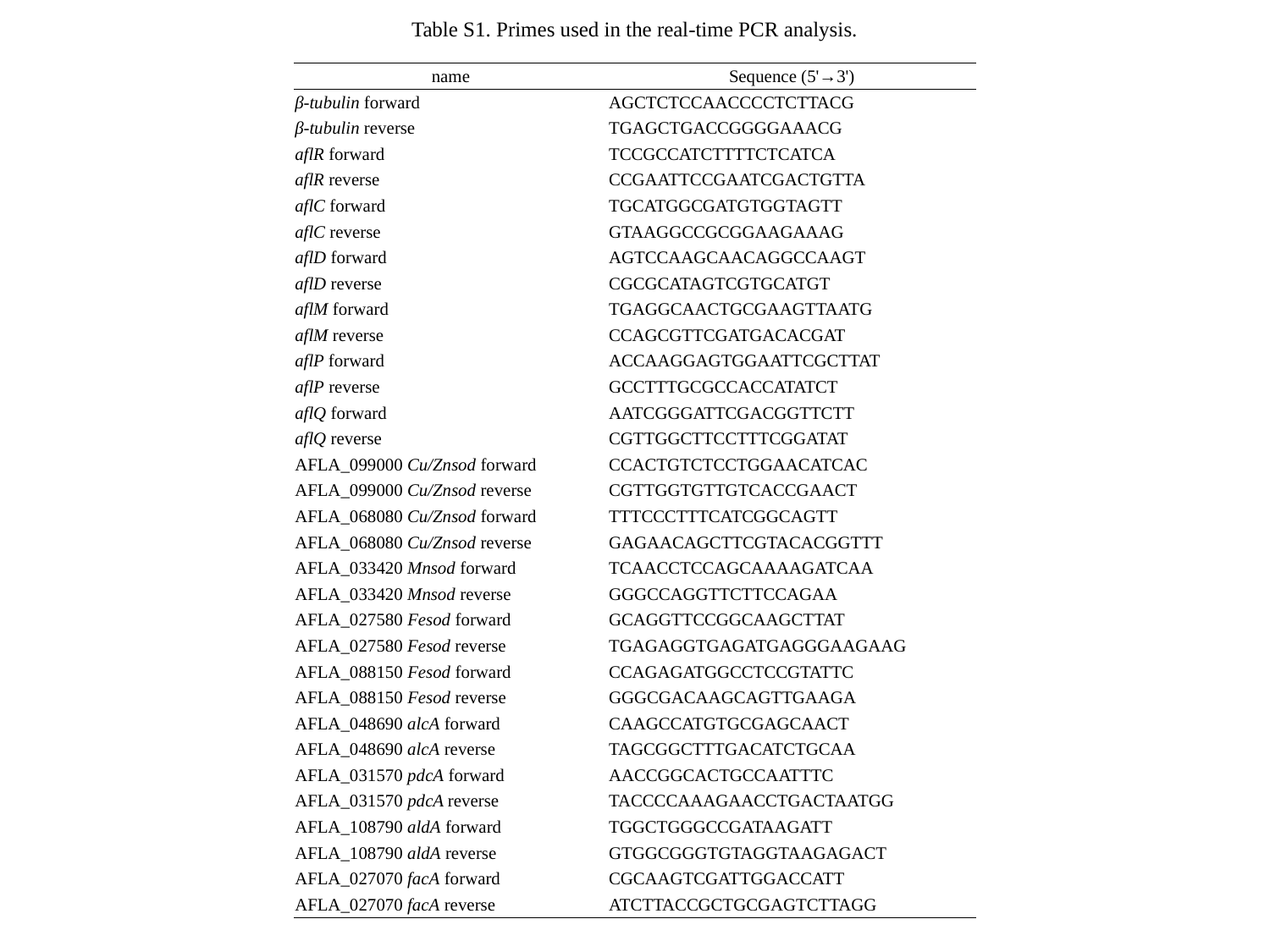

Table S1. Primes used in the real-time PCR analysis.
| name | Sequence (5'→3') |
| --- | --- |
| β-tubulin forward | AGCTCTCCAACCCCTCTTACG |
| β-tubulin reverse | TGAGCTGACCGGGGAAACG |
| aflR forward | TCCGCCATCTTTTCTCATCA |
| aflR reverse | CCGAATTCCGAATCGACTGTTA |
| aflC forward | TGCATGGCGATGTGGTAGTT |
| aflC reverse | GTAAGGCCGCGGAAGAAAG |
| aflD forward | AGTCCAAGCAACAGGCCAAGT |
| aflD reverse | CGCGCATAGTCGTGCATGT |
| aflM forward | TGAGGCAACTGCGAAGTTAATG |
| aflM reverse | CCAGCGTTCGATGACACGAT |
| aflP forward | ACCAAGGAGTGGAATTCGCTTAT |
| aflP reverse | GCCTTTGCGCCACCATATCT |
| aflQ forward | AATCGGGATTCGACGGTTCTT |
| aflQ reverse | CGTTGGCTTCCTTTCGGATAT |
| AFLA\_099000 Cu/Znsod forward | CCACTGTCTCCTGGAACATCAC |
| AFLA\_099000 Cu/Znsod reverse | CGTTGGTGTTGTCACCGAACT |
| AFLA\_068080 Cu/Znsod forward | TTTCCCTTTCATCGGCAGTT |
| AFLA\_068080 Cu/Znsod reverse | GAGAACAGCTTCGTACACGGTTT |
| AFLA\_033420 Mnsod forward | TCAACCTCCAGCAAAAGATCAA |
| AFLA\_033420 Mnsod reverse | GGGCCAGGTTCTTCCAGAA |
| AFLA\_027580 Fesod forward | GCAGGTTCCGGCAAGCTTAT |
| AFLA\_027580 Fesod reverse | TGAGAGGTGAGATGAGGGAAGAAG |
| AFLA\_088150 Fesod forward | CCAGAGATGGCCTCCGTATTC |
| AFLA\_088150 Fesod reverse | GGGCGACAAGCAGTTGAAGA |
| AFLA\_048690 alcA forward | CAAGCCATGTGCGAGCAACT |
| AFLA\_048690 alcA reverse | TAGCGGCTTTGACATCTGCAA |
| AFLA\_031570 pdcA forward | AACCGGCACTGCCAATTTC |
| AFLA\_031570 pdcA reverse | TACCCCAAAGAACCTGACTAATGG |
| AFLA\_108790 aldA forward | TGGCTGGGCCGATAAGATT |
| AFLA\_108790 aldA reverse | GTGGCGGGTGTAGGTAAGAGACT |
| AFLA\_027070 facA forward | CGCAAGTCGATTGGACCATT |
| AFLA\_027070 facA reverse | ATCTTACCGCTGCGAGTCTTAGG |
